# Precocious Puberty and Normal Variant Puberty: Definition, etiology, diagnosis and current management

**DOI:** 10.4274/jcrpe.v1i4.3

**Published:** 2010-12-08

**Authors:** Merih Berberoğlu

**Affiliations:** 1 Ankara University School of Medicine, Department of Pediatric Endocrinology, Ankara, Turkey; +90-312-595 64 34+90-312-319 14 40merihbtr@yahoo.comAnkara University School of Medicine, Department of Pediatric Endocrinology, Ankara, Turkey

**Keywords:** Precocious puberty, premature thelarche, premature pubarche

## Abstract

This review describes several aspects of the management of precocious puberty (PP) and variants in girls and boys.

PP is characterized by early pubertal changes, acceleration of growth velocity and rapid bone maturation that often result in reduced adult height. Onset of pubertal signs before the age of 8 years in girls and 9 years in boys should always be evaluated carefully. The main principles of therapy are to stop the progression of secondary sex characteristics and menses (in girls), to increase final adult height, to promote psychosocial well-being, and to treat the underlying cause if known.

**Conflict of interest:**None declared.

Precocious puberty (PP) is defined as the development of pubertal changes, at an age younger than the accepted lower limits for age of onset of puberty, namely, before age 8 years in girls and 9 years in boys. PP is responsible for early progression of secondary sexual characteristics, rapid bone maturation, reduced final height, inappropriate body appearance and psychological behavioral abnormalities. Indications for treatment are based on the progression rate of puberty, advancement of bone age, predicted adult height and psychological evaluation. This paper will focus on central precocious puberty (CPP), normal variant puberty, and current management principles for these conditions.

According to the classical definition, onset of secondary sexual characteristics as Tanner Stage 2 in a girl before age 8 years, or in a boy before age 9 years is defined as PP. However, according to two studies from USA, the onset of puberty may be as early as 7.7 years in girls and as early as 7.6 years in boys.^1^ Although there is no consensus, breast development in girls aged between 7-8 years is defined as the “gray zone” and this clinical phenomenon has been referred to as “accelerated puberty, early normal puberty, or rapid progressive thelarche variant”. Majority of these girls do not require treatment and reach a final height which is appropriate for their target height.

It may be hard to diagnose PP in a child with minimal symptoms. Especially in girls, PP diagnosis should be confirmed by showing increase in gonadotropin and/or sex steroids levels, accelerated somatic development and bone age advancement. Follow-up of the early signs of sexual development is also important. If these signs do not progress at follow-up, early breast development can be accepted as a normal variant.

PP may be classified as gonadotropin dependent, progressive (central/true PP) or gonadotropin independent (peripheral/pseudo PP). The hypothalamus, pituitary and gonad (HPG) axis is active in both physiological puberty and central true PP. However, pseudo- or peripheral PP is independent of gonadotropin secretion and there is no activation of the HPG axis. The source of sex steroids is exogenous and/or endogeneous in this subgroup. The etiological classification of PP is seen in Table 1.

**Table 1:**
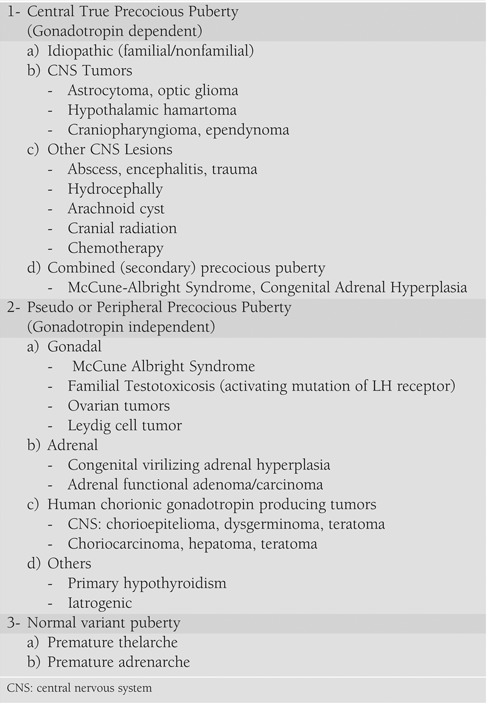
Etiological classification of precocious puberty

## CENTRAL PRECOCIOUS PUBERTY (CPP)

CPP is due to early maturation of the HPG axis. The frequency of CPP ranges between 1/5000-1/10,000.^2, 3^ It is more common in girls. Female/male ratio changes between 3/1 and 23/1.^4^ It is reported to be more frequent in adopted children in developed countries.^5^

Although the large majority of CPP is idiopathic, organic lesions as well as environmental factors and substrates that mimick hormones (endocrine disrupting chemicals) may have estrogen activity or may cause an increase of endogenous estrogen secretion. Cosmetic products, foods and some pharmacological insecticides can disturb the HPG axis and result in PP.^6, 7^

Many researchers have shown that childhood obesity may be associated with early menarche in girls. Increased caloric intake and consumption of fast foods, decreased physical activity and prolonged television watching duration are the factors that may cause obesity.^6, 8^

The relationship between intrauterine growth retardation, premature adrenarche and early menarche has also been brought to attention in recent years.^9^

Although the frequency of central true PP is rare, the progressive decrease in age of breast development noted particularly in the second half of the 20^th^ century, drew attention to a need for reconsideration of the age of normal puberty. Endocrine disturbances, obesity and low birth weight, are among factors which have been held responsible for increased frequency of thelarche variants in PP distribution, so that there seems to be a need for new definitions in the 21^st^ century.

The etiology of CPP cannot be established in many cases in girls and the condition is accepted as idiopathic. Cerebral lesions are found in a small minority of the cases. It is known that the risk for CPP is increased in patients with neurofibromatosis type 1, hydrocephaly, meningomyelocele, neonatal encephalopathy and in those exposed to low-dose cranial radiation. 

While girls are prone to PP, delayed puberty is more frequent in boys. However, the risk of CPP due to organic causes is higher in boys. One of the most common observed pathologies is hypothalamic hamartoma. However, central nervous system abnormalities, especially lesions in the hypothalamic region may not be easily shown by imaging methods.

Hypothalamic hamartomas are congenital, non-neoplastic, and tumor-like lesions. Some hamartomas present with gelastic seizures and are resistant to anticonvulsant treatment.^10^ The suspicious symptoms for hypothalamic hamartoma can be listed as:^5^

1. Occurrence of PP before 2 years (generally 4 years) of age

2. PP being gonadotropin dependent

3. Presence of isointense tumor on magnetic resonance imaging with gadolinium

4. Negative tumor markers for pseudo PP.

Hamartomas are more frequent in males. CPP caused by hamartoma occurs earlier, even before 4 years, when compared to idiopathic and other organic etiologies in both sexes.

The tissue in hypothalamic hamartomas includes GnRH neurons. These ectopic neurons are functional and secrete GnRH episodically. Transforming growth factoralpha receptors that express astrogial cell are also found in hamartomas. The frequency of clinical CPP with hypothalamic hamartomas before 4 years of age is 10%.^2^

The risk of CPP is higher in cases diagnosed as malignancy and who have received radiotherapy and chemotherapy. However, if growth hormone deficiency is associated, the acceleration in growth may not be evident.

A similar condition is present in neurofibromatosis type 1. The first clinical symptom in children with optic glioma would be CPP. If it is not diagnosed, it may progress to complete blindness.

Other causes that should be considered are the gene mutations that cause PP. The family investigations especially on recently defined GRP54 receptor gene in idiopathic cases and its ligand kisspeptin, and GnRH receptor gene will increase the information in this field.^11^

## COMBINED SECONDARY CENTRAL PRECOCIOUS PUBERTY

Acceleration in somatic development and advancement in bone age are inevitable findings when the patient is exposed to high level of sex steroids in a long period. This results in an earlier puberty and HHG axis matures depending on the somatic development, rather than chronological age. The most common etiology for this situation is the congenital adrenal hyperplasia. Testotoxicosis and McCune-Albright syndromes were also defined as rare causes.^5^ All peripheral pseudo PP cases without management would become complicated when CPP is superimposed. The onset of CCP is related to the degree of somatic development. Generally it starts when bone age reaches 10-13 years. However, in some patients it starts just 1 month after the onset of the treatment for pseudo PP.^12^ Secondary CPP is permanent and requires treatment.

## PSEUDO- OR PERIPHERAL PRECOCIOUS PUBERTY (PPP)

In girls one of the most common etiologies of PPP is ovarian follicular cysts. These cysts may cause vaginal bleeding in younger girls. Pelvic ultrasonographic evaluation is very useful in such occasions. In some cases, especially with small cysts, there may be regression by time, and estrogen level decreases. These cases are generally the ones who were followed as premature menarche in the past. Although it is not justified, premature menarche cases are not accepted as normal variant and their follow-up for PPP is recommended.

Granulosa- theca cell cysts or tumors are generally bigger than follicular cysts.

The classical triad of McCune-Albright Syndrome includes café-au-lait spots, poliostatic fibrous dysplasia and PPP. It occurs due to the mutation that activates the gene encoding Gs protein-alpha subunit. It should be kept in mind in children who have recurrent follicular cysts and irregular vaginal bleeding even without other signs. It may have incomplete forms. The other endocrine organ hyperfunctions (hyperthyroidism, Cushing syndrome, acromegaly, and hypophosphatemic rickets) should be investigated. Bone scintigraphy is necessary in these cases.

LH receptor activating mutations (familial testotoxicosis) are autosomal dominant rare diseases in male children. It is characterized by symmetrical testicular enlargement. McCune Albright syndrome is rare in boys. HCG secreting tumor is another cause of PPP in boys. It may be located in organs other than gonads, such as liver due to hepatoblastoma, pineal region, brain or mediastinum. This results in testicular enlargement and elevation in serum testosterone levels. Leydig cell tumors are generally benign and cause unilateral testis enlargement.^2^ PPP etiologies in both sexes which were stated up to here are isosexual.

Virilizing diseases that arise from adrenal gland, especially congenital adrenal hyperplasia, are the most common PPP etiological factors in childhood. Although it causes isosexual PPP in boys, it results in heterosexual PPP in girls.

**Chronic primary hypothyroidism**

Significant TSH increase in some prepubertal children causes an increase in FSH level and results in occurrence of pubertal signs. Early breast development in girls and mild testicular enlargement in boys compose the clinical signs. Acceleration in somatic development is not seen. Increase in prolactin level and galactorrhea may accompany the pubertal signs. The treatment of hypothyroidism causes a recovery.^2^

## PREMATURE THELARCHE

Premature thelarche typically starts before 2 years of age and breast development is isolated. It may be unilateral. Somatic development is not accelerated. The bone age is not advanced. Breast development may be cyclical in relation to blood estrogen levels. The situation regresses by time. The same regression is not observed in cases that start after 2 years of age and have significant glandular structure. Primary hypothyroidism should be ruled out in these cases.

The follow-up for PP is necessary in cases that were defined as thelarche variant. Premature thelarche which starts after 2 years of age may progress to CPP.^2^ Advancement in bone age, ovarian enlargement and higher estrogen levels may accompany premature thelarche in borderline cases.

The physiologic baseline event in premature thelarche is the increase in FSH level. Inhibin B secreted from granulose cells is thought to be responsible for this increase.^13^ FSH response is the foreground both in night pulses and peak response to GnRH stimulus. LH response together with FSH may increase to higher levels in premature thelarche cases that start before 2 years of age, and it may interfere with CPP. This phenomenon has been termed as “mini-puberty”.^14^ However, ovaries and uterus are within prepubertal sizes, but these measurements must be performed by an experienced physician. The follow-up of progression rate and growth tempo are important in these cases. Another point which should be kept in mind is, that some of the premature thelarche cases are due to exposion of estrogenic environmental pollution.^6^

**Premature adrenarche/pubarche**

Adrenarche is the increase of pubertal adrenal androgens. It may be seen in both sexes in normal children between 6-8 years of age. Generally pubarche, that is, genital and axillary hair, does not accompany this situation. However, adrenarche is associated with pubarche in some children, and this is the most innocent form of the premature pubarche. It is important to distinguish this situation from pathologic etiologies.

Adrenarche occurs as a result of increased secretion of androgens from zona reticularis from the adrenal cortex. It results in an increase of serum DHEA and its metabolite sulfate due to an increase in 17, 20 liyase and 17 alpha hydroxylase activities. Increase in serum level of DHEA-S over 40 μg/dl is the biochemical indicator of the adrenarche. This increase may be exaggerated in some cases, and may increase up to 200 μg/dl. In a recent study it was shown that androgen receptor gene activity increase due to decreased receptor gene methylation pattern can be seen in some cases.^15^

Congenital adrenal hyperplasia or adrenal tumors, in which androgen production is increased, are the pathologies which should be ruled out initially in premature pubarche. Some partial enzyme deficiencies such as 21-OH and 3β OHD deficiency may manifest first with premature pubarche. Both somatic growth and advancement in bone age are accelerated in these cases; so that DHEA-S measurement should be performed in addition to testosterone and 17OHP. Although DHEA-S is increased in isolated premature pubarche, testosterone and 17OHP levels are in normal ranges. Sometimes, ACTH stimulation test is necessary in borderline cases for diagnosis. Onset of puberty in cases with premature adrenarche is generally expected in normal time. Final heights must be compatible with their genetics. However, prepubertal growth tempo may slightly be increased. This condition does not affect the onset and progression of normal puberty. In some conditions, gonadarche (HPG axis activation) may accompany adrenarche, so that, the follow-up of cases at 4-6 month intervals is suggested.

Recently the association of ovarian hyperandrogenism (polycystic ovary syndrome), hyperinsulinism and dyslipidemia observed in some girls with premature pubarche has gained importance.^16, 17^ Premature pubarche may be the earlier sign of metabolic syndrome in childhood. Hyperinsulinism in these cases may be the result of diminished fetal growth rate and intrauterine growth retardation.^9, 18, 19, 20^

There is risk for early menarche in some cases with onset of pubarche between 7-8 years. The predicted adult height is also affected in some cases. It has been observed that 45% of girls with premature pubarche developed polycystic over syndrome in time.^2^ Although some cases have low birth weight, some may be in normal weight ranges.^2^ Family history of girls with premature pubarche should be evaluated for metabolic syndrome and these girls should be followed for this risk independent of their birth weight. Today the question of which patients in gray zone carry this risk is not clarified, and is a research subject. The audit of hyperinsulinism and increase of insulin sensitivity in low-birth weight patients may prevent early menarche in some cases in the future.^21^

## CLINICAL SIGNS AND DIAGNOSIS

PP is associated with accelerated growth, advanced bone age, development of secondary sex characteristics and early closure of epiphysis. Defining the etiologic cause is important for the management of the underlying disease. History should be sought for information about the onset of the signs, progression rate, and growth tempo in the last 6-12 months, presence of secondary sex characteristics (acne, oily skin, erection, night ejaculation and vaginal bleeding) in addition to the presence of pubertal signs. History of PP in family supports the diagnosis of familial forms. Pubertal staging should be performed according to Tanner-Marshall method on physical examination, and anthropometric evaluations should be defined by measurement of weight, height and body proportions. All old and new data should be marked on growth chart. Growth velocity per year must be calculated. If there is not any data for the past, patient should be followed prospectively for at least 6 months. Growth velocity is more than 75^th^ percentile in most patients with CPP. Bone age should be determined by left hand and wrist X-ray. If bone age is advanced more than 2SD for chronological age it is unlikely the child has a normal variant of pubertal development.^5^ If it is possible, Δ bone age/ Δ chronological age must be calculated. If this ratio is greater than 1.2, it is in favor of progressive CPP.

If testis volume is smaller than 4 ml in the presence of secondary sex characteristics in a boy, this PP is probably caused by adrenal pathologies. Asymmetric testicular enlargement is observed in McCune-Albright syndrome and Leydig cell tumor cases, whereas bilateral testicular enlargement is common in testotoxicosis, hCG secreting tumors and CPP cases. Testis enlargement is in moderate degree, and never exceeds Tanner 3 in testotoxicosis and hCG secreting tumors. Cases with only one of the pubertal signs, without accelerated growth and advanced bone age can be accepted as normal variant, but should be followed-up.

The first endocrinological evaluation includes the measurement of gonadotropins (LH, FSH) and related sex steroids. Old radioimmunassay kits are not definitive in baseline gonadotropin evaluation for differentiation between CPP and PPP. The patient must be evaluated by GnRH stimulation test. However, third generation sensitive immunochemilumimetric (ICMA) measurements are sensitive at basal evaluation, but GnRH test is still the most important differential diagnostic test. Although FSH response is dominant in premature thelarche, LH is the dominant gonadotropin in CPP patients. LH and FSH are suppressed or at prepubertal levels in PPP cases. 0 and 30 minute samples may be sufficient on intravenous GnRH test.^5^ There is no evidence for a superior response in prolonged tests.^5^ In premature thelarche cases that are younger than 2 years old; LH may increase in addition to FSH, but dominant response is still FSH. There is a pubertal response of LH to GnRH in CPP patients.^5^ Organic/idiopathic etiological evaluation can not be done by only hormonal levels for CPP.^22^

The diagnosis of CPP should not be based on hormonal data and it must be combined with clinical signs and follow-up. Basal plasma testosterone level in boys increase both in CPP and PPP, but it is much higher in PPP patients. The importance of oestrogen level is limited in girls with CPP, because in many affected girls it has been shown in low ranges.^3^ The other endocrinological evaluations may include thyroid tests, 17OHP level and hCG measurement depending on the clinical signs.

Pelvic ultrasonography is another test that should be performed in girls. Ovarian and uterine sizes should be compared with reference levels. Increase in ovarian volumes are important especially in CPP cases. Bilateral enlarged ovaries can be determined in CPP patients. Uterine volume is pubertal in all CPP cases.^23^ Although structure of ovaries (microcystic/macrocystic/ follicular), follicule diameter, fundus/ cervix ratio, uterine length and endometrium thickness are important parameters, many experts reported that these were not determinant in differential diagnosis between premature thelarche and CPP. Ovaries may be asymmetrically enlarged in girls with gonadal PPP. Ovaries show microcystic structure on ultrasonography in prepubertal ages. Uterine shape is tubuler, endometrium is thin. Ovaries are smaller than 2 ml, and uterine length is smaller than 4 cm.^23^ By contrast ovaries are macrocystic/follicular on pubertal ultrasonography. Uterine is in “pear” shape and endometrium is thickened.

**Figure 1:**
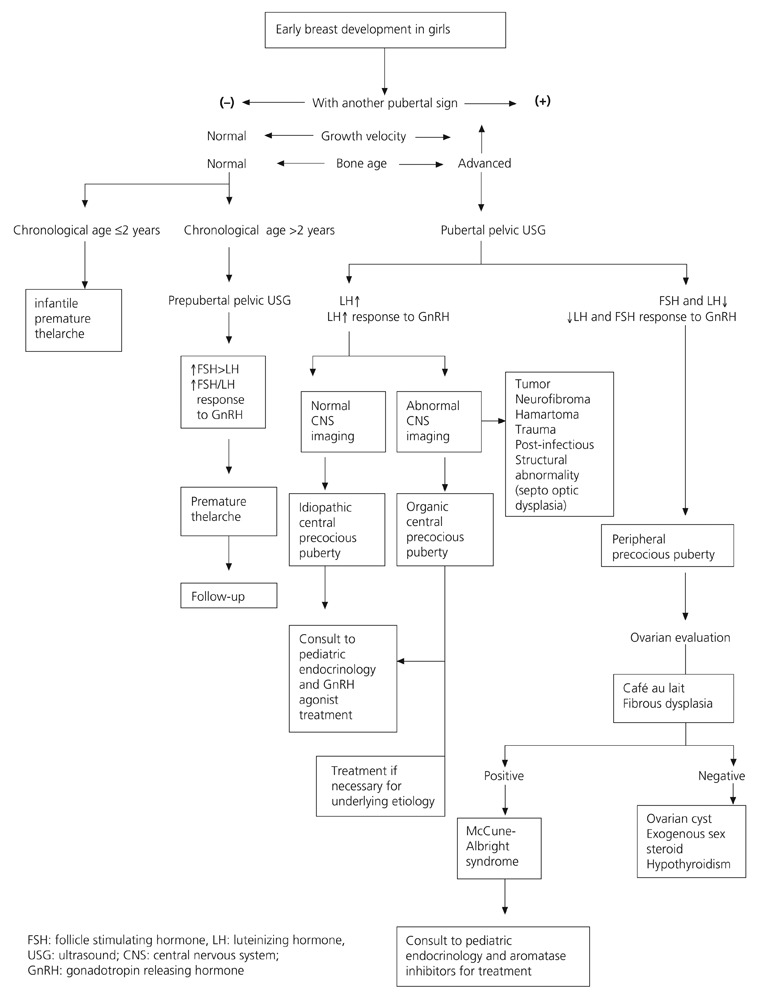
Flow chart for breast development in girls

**Figure 2:**
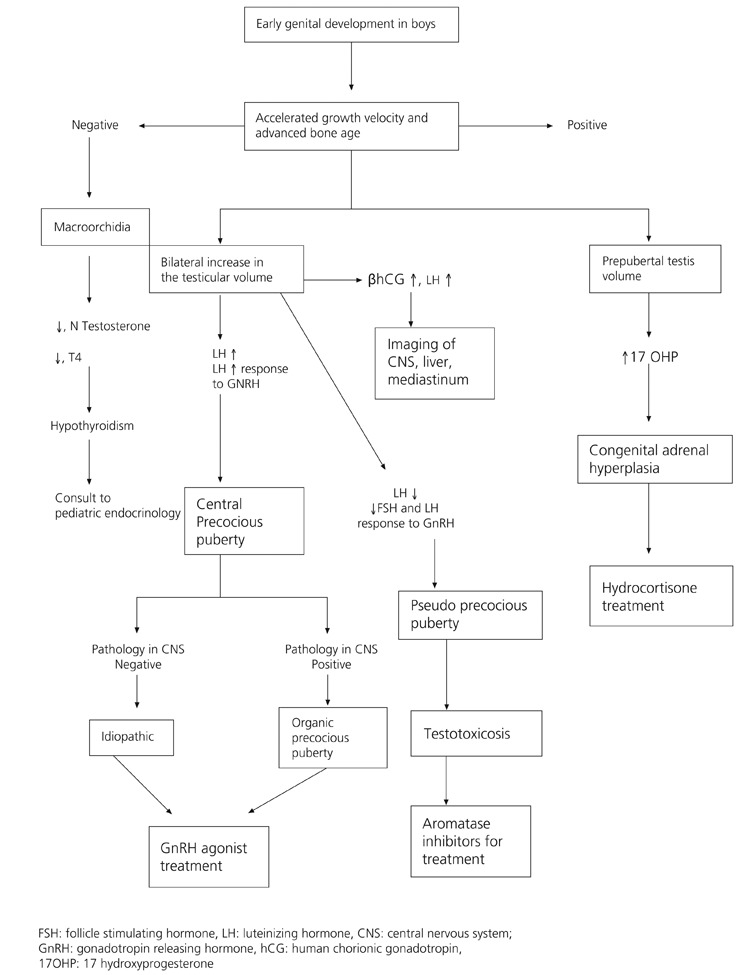
Flow chart for precocious puberty in boys

Cranial and pituitary magnetic resonance imaging (MRI) should be performed to rule out organic CPP. Although there has been a suggestion to perform MRI in only male CPP cases or in girls below 6 years old, today it is accepted that MRI should be done for all cases because intracranial tumors may cause CPP in all ages.^2, 5, 8, 24^ However, imaging needs to be repeated periodically in cases who are below 4 years and were reported to be normal. It is important that the MRI images be evaluated by expert radiologists. Flow charts for PP in boys and girls are shown on Figure 1 and Figure 2.

## CURRENT MANAGEMENT APPROACHES

There is no consensus about the treatment indications for CPP. Treatment options may depend on the presence of psychological/behavioral disorders, anxiety about height and probability of early menarche. Families and physicians should discuss the risks and decide on the treatment together. Although psychological influences of PP have not been investigated sufficiently, there are some case reports about sexual abuse and early pregnancy. Psychological indication should be based on the patient individually.

There is not a hesitance about the initiation of treatment for cases younger than 7 years old. However, especially for patients between 7-8 years, rapid advancement in bone age, more than 1 SD loss in predicted adult height and probability of menarche before 10 years old compose the concrete criteria for treatment. However, there is not a treatment indication for slowly progressive CPP without loss in height potential.^25^ GnRH analogues have been used for suppressing HPG axis since 1981. Their effects depend on desensitization and downregulation of GnRH receptors. They are safe and have minimal side effects.

The most commonly used one, which has also been added to indication list in our country, is the leuprolide acetate depot form. Recently, subcutaneous form which is less painful and can be applied at 3 to 4 week intervals is on market. In the first months of the treatment, there can be exacerbation in signs which may result in vaginal bleeding in girls, but this effect is infrequent than expected. Some minor menopausal signs have been defined. The depression that was reported in adults has not been defined in children yet. Although there have been studies concerning body proportions, obesity and psychological effects, they are not evidence-based. The suggested regimen is 0.3 mg/kg/28 days. The experience with 3 month depot form is limited.

Effective GnRH agonist treatment can be succeeded only by efficient Gn suppression. Bone age, growth velocity, uterine and ovarian sizes and breast development should be followed closely during the treatment. The treatment does not affect the pubic hair. LH suppression should be checked by iv GnRH test at 3-6^th^ month of the treatment. Follow-up with oestrogen levels is not recommended.

HPG axis returns to its previous situation after the cessation of the treatment. The most frightening side effect, especially in patients who received growth hormone for suppressed growth due to GnRH agonist treatment, is the development of polycystic ovaries.^26^ There is not any information about the long term follow-up of those patients in the literature. However, we have information about the final height. Results are slightly better in boys when compared to girls.^5^ The use of GnRH analogues, especially after 7.5 years, does not have effect on final height.^23^ However, final height results are better when compared to untreated PP patients. 75% of patients reach their genetic potential, and height is > 150 cm in 90%.^28^ Agonist treatment does not cause osteopenia or osteoporosis. Body proportions were reported to be better when compared to untreated patients.^5^

A new treatment option, is histrelin, subcutaneous implant of GnRH agonist.^30^ It was developed to decrease the monthly hospital visits. There is a new research on using both agonist and short acting antagonist to suppress the exacerbation in the first months, but all are experimental studies.

PPP treatment options are anti-oestrogens like tamoxifen or aromatase inhibitors such as anastrozole.^32^ Pamidronate constitutes the current option in the treatment of fibrous dysplasia in McCune Albright Syndrome.^3^

## CONCLUSION

There are three questions to be answered when faced with PP. 

1. Is PP a normal variant or an abnormal sign?

2. If it is abnormal, is it central or peripheral, and if it is peripheral, is it adrenal or gonadal?

3. If it is central, is it idiopathic or related to an intracranial pathology? Is there an indication for treatment?

If these questions are answered, data about approach and management are evidence-

based and clear. Treatment indication in intermediate forms depends on cost benefit ratio. Close follow-up of all patients constitutes the main aspects of the management.
